# Non-steroid anti-inflammatory drugs, prostaglandins, and cancer

**DOI:** 10.1186/2045-3701-3-8

**Published:** 2013-02-06

**Authors:** Viola Allaj, Changxiong Guo, Daotai Nie

**Affiliations:** 1 Department of Medical Microbiology, Immunology, and Cell Biology, Southern Illinois University School of Medicine and Simmons Cancer Institute, Springfield, IL, 62794, USA; 2 Department of Medical Microbiology and Immunology, Southern Illinois University School of Medicine, PO Box 19626, Springfield, IL, 62794, USA

## Abstract

Fatty acids are involved in multiple pathways and play a pivotal role in health. Eicosanoids, derived from arachidonic acid, have received extensive attention in the field of cancer research. Following release from the phospholipid membrane, arachidonic acid can be metabolized into different classes of eicosanoids through cyclooxygenases, lipoxygenases, or p450 epoxygenase pathways. Non-steroid anti-inflammatory drugs (NSAIDs) are widely consumed as analgesics to relieve minor aches and pains, as antipyretics to reduce fever, and as anti-inflammatory medications. Most NSAIDs are nonselective inhibitors of cyclooxygenases, the rate limiting enzymes in the formation of prostaglandins. Long term use of some NSAIDs has been linked with reduced incidence and mortality in many cancers. In this review, we appraise the biological activities of prostanoids and their cognate receptors in the context of cancer biology. The existing literature supports that these lipid mediators are involved to a great extent in the occurrence and progression of cancer.

## Introduction

Dietary fat is an important energy source. Fatty acids that are produced from catabolism of fats compose an important aspect of a healthy diet. They are subcategorized into saturated (lack double bonds) and unsaturated (contain double bonds) fatty acids. Fatty acids, including polyunsaturated fatty acids (PUFA), are usually stored in phospholipids or triglycerides. Essential fatty acids are necessary polyunsaturated fats that the human body is unable to synthesize and must obtain through the diet. Two families with opposing effects belong to this category of fatty acids: linoleic acid (omega-6) and alpha-linolenic acid (omega-3), which are the precursors of arachidonic acid (AA) and eicosapentanoic acid respectively.

**Figure 1:**
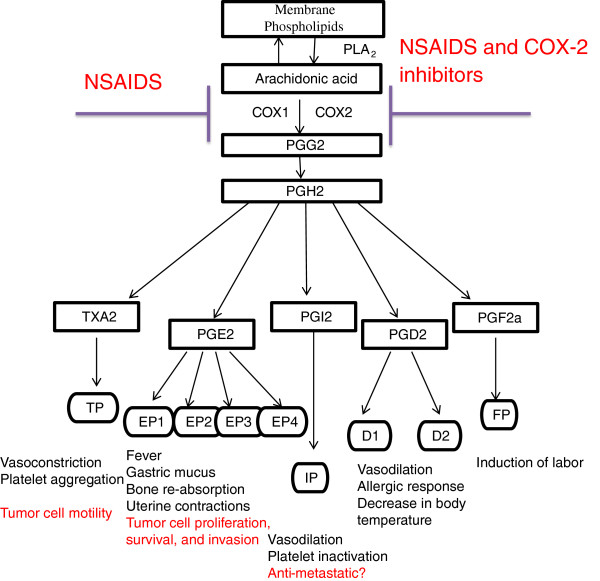
**Biosynthesis and activities of prostaglandins and sites of NSAIDs actions.** Cyclooxygenase metabolism of arachidonic acid can lead to the formation of prostaglandins that exert a variety of biological activities through their respective cognate receptors. The involvement of prostanoid receptors in cancer is also shown. Abbreviations: COX, cyclooxygenase; PG, prostaglandin; PLA_2_, phospholipase 2; TXA_2_, thromboxane A_2_; TP, thromboxane A_2_ receptor; EP, prostaglandin E_2_ receptor; IP, prostacyclin (PGI_2_) receptor; DP, prostaglandin D_2_ receptor; FP, prostaglandin F_2_ receptor; NSAIDs, non-steroid anti-inflammatory drugs.

In humans, cellular AAs are mainly released from membrane phospholipids by phospholipase A_2_ and phospholipase C. AAs can also be cleaved from diacylglycerol and is a minor product of linoleic acid (LA) metabolism. Most obligate carnivores, however, cannot synthesize AA from LA and must obtain AA from dietary sources. Arachidonic acid can be metabolized through cyclooxygenase (COX), lipoxygenase (LOX), or epoxygenase mediated pathways to form a variety of biologically active lipids, termed as eicosanoids. LOX-derived metabolites include hydroperoxyeicosatetraenoic acids (HpETE), leukotrienes (LT), and lipoxins (LX) [1]. The COX pathway produces prostaglandin (PG) G_2_ and prostaglandin H_2,_ which is further converted into other prostaglandins. The major prostanoids synthesized from COX include prostaglandin E_2_, prostaglandin D_2_, prostacyclin I_2_, prostaglandin F_2a_ and thromboxane A_2_ (TXA_2_) (Figure 1). Prostanoids are extensively studied for their involvement in a long list of adverse health conditions, including cancer, inflammation, thrombosis, arthritis and atherosclerosis.

Non-steroid anti-inflammatory drugs (NSAIDs) are widely consumed as analgesics to relieve minor aches and pains, as antipyretics to reduce fever, and as anti-inflammatory medications. Most NSAIDs are nonselective inhibitors of cyclooxygenases (COX), the rate limiting enzyme in the formation of prostaglandin H_2_. Therefore, NSAIDs can reduce the formation of various prostanoids. A number of epidemiological studies have linked the long term use of some NSAIDs, especially aspirin, with reduced cancer incidence and most significantly, with reduced cancer mortality [2-4]. This paper aims to give a brief overview of the effect of cyclooxygenases and the prostanoid signaling in the initiation, progression and treatment of cancer and provide an appraisal of NSAIDs utility in cancer prevention and treatment.

## Cyclooxygenases

Currently, three known isoforms of COX exist: COX-1, COX-2, and COX-3. COX-1 and COX-2, also known as prostaglandin-endoperoxide synthase 1 and 2 respectively, catalyze the rate limiting step of prostaglandin synthesis. COX-1, encoded by the PTGS1 gene, is constitutively expressed in most mammalian tissues and appears to regulate normal physiological functions, including the maintenance of vascular homeostasis, mediation of allergic and immune responses, and stimulation of gastric mucosa production. COX-2, encoded by PTGS2 shares 81% homology with COX-1, is usually absent from healthy tissue and is transiently induced by pro-inflammatory stimuli, growth factors, cytokines, and tumor promoters to increase the rate of prostaglandin formation after tissue injury [1]. COX-3 is an alternately spliced variant of COX-1. Also encoded by the PTGS1 gene, COX-3 proteins retain an intron and a frame shift mutation, resulting in non-functional proteins in mice and humans [5].

When an appropriate PUFA binds to the COX active site, COX catalyzes the oxygenation of the substrate into 5-R,6-R’,(1*R*,4*S*)-2,3-dioxabicyclo[2.2.1]heptane through a series of carbon radical intermediates. Both COX-1 and COX-2 primarily, but not exclusively, oxygenate AAs into prostaglandin G_2_s (PGG_2_)-at least three other minor products exist. PGG_2_s are then reduced to prostaglandin H_2_s (PGH_2_) by peroxidases. PGH_2_ is subsequently converted into biologically functional molecules-including prostaglandin E_2_ (PGE_2_), which induces fever and stimulates gastric mucus production, bone re-absorption and uterine contractions; prostaglandin D_2_ (PGD_2_), which mediates vasodilatation, allergic response, and decreases in body temperature; prostacyclin (PGI_2_), which potently induces vasodilatation and inhibits platelet activation; and thromboxane (TXA_2_), which functions in contrast to PGI_2_ and potently induces vasoconstriction and platelet aggregation (Figure 1).

COX-1 and COX-2 also catalyze the oxygenation of other PUFAs, including dihomo-γ-linolenic acid (DGLA) to form the series-1 prostaglandin and thromboxane precursor PGH_1_ and series-1 thromboxane TXA_1_, eicosapentaenoic acid (EPA) to form series-3 prostaglandin precursor PGH_3_, and possibly linolenic acids to form 9- and 13-hydroxyoctadecadienoic acids (HODE) [6,7].

The COX enzymes are the primary targets of NSAIDs, including common over the counter medications such as aspirin, ibuprofen, and naproxen. By inhibiting the production of PGs, these drugs suppress the pathways that mediate inflammation, pain and fever. Long term use of some of these drugs appears to also offer limited protection against acute cardiac events, Alzheimer’s disease, and neoplastic diseases, especially colorectal neoplasms. But the medications can potentially interfere with the production of gastrointestinal mucosa, significantly increasing the risk of gastrointestinal related side effects, including dyspepsia, abdominal pain and occasional perforations. More recently developed COX-2 specific inhibitors, such as rofecoxib (Vioxx), are not associated with GI related side effects, but appear to, along with ibuprofen, significantly increase the chances of acute myocardial infarction in already at risk patients-enough of a risk to prompt Merck to voluntarily withdraw Vioxx from market [1].

## Prostanoid signaling

Prostanoids usually act within the tissue, where they are synthesized, via a carrier-mediated process [8] to activate the membrane receptors [9-11], or in some cases may interact with nuclear receptors [12]. The membrane receptors for prostanoids are termed P receptors, with a preceding letter indicating the natural prostanoid to which each receptor is most sensitive, i.e. DP, EP, FP, IP and TP for PGD_2_, PGE_2_, PGF_2_, PGI, and TXA_2_, respectively [13,14] (Figure 1). The membrane receptors for prostanoids are mainly heterotrimeric G protein coupled receptors (GPCR). Below is a brief overview of GPCRs for prostanoid signaling.

EP: PGE_2_ regulates diverse biological processes, including cell growth, inflammation, reproduction, sodium homeostasis and blood pressure [15]. Its biological effects are complex and often opposing; vasodilation in arterial and venous systems but constriction of smooth muscle in the trachea, gastric fundus and ileum [16]. Four EP receptor subtypes (EP1-4) have been cloned and characterized [17-21]. The mRNAs for all EPs are widely expressed. Although these four receptors uniformly bind PGE_2_ with high affinity, when examined on the basis of amino acid homology, they are not as closely related to each other as to other prostanoid receptors that use similar signaling mechanisms [14]. EP2 and EP4 are more closely related to IP and DP receptors, whereas EP1 and EP3 are more closely related to TP and FP receptors.

The EP1 was originally described as a smooth-muscle constrictor. It plays important roles in neuronal functions. The cloned human EP1 receptor cDNA encodes a 402-amino-acid polypeptide that signals via increased inositol-3-phosphate (IP3) generation and increased intracellular Ca^2+^ moblization [17,22].

The human EP2 receptor cDNA encodes a 358-amino-acid polypeptide that signals through increased cAMP [21,23]. EP2 receptor plays an important role in female reproduction, vascular hypertension and tumorigenesis [24]. The EP2 may also be an important target for treating asthma by promoting bronchodilation [25]. The EP2 is distinguished from the EP4 by its relative insensitivity to the EP4 agonist PGE1-OH [23]. EP2 mRNA is most abundant in the uterus, lung, and spleen, with only low levels of expression in the kidney [23,26]. It is expressed at much lower levels than EP4 mRNA in most tissues [27].

The EP3 receptors generally act as constrictors of smooth muscle [13]. It is associated with fever, gastric mucosal protection, pain hypersensitivity, kidney function and anti-allergic response. EP3 has at least nine alternatively spliced variants defined by unique C-terminal cytoplasmic tails [28]. These splice variants encode proteins with predicted molecular masses of between 40 and 45 kDa [23]. The EP3 splice variants bind PGE_2_, agonists MB28767 and sulprostone with similar affinities as EP3 [14]. All of the splice variants uniformly and potently inhibit cAMP generation via a pertussis toxin-sensitive Gi-coupled mechanism; however, Ca^2+^-dependent signaling mechanisms appear to be differentially activated by different C-terminal tails [29-31]. The physiological significance of these different C-terminal splice variants may play a role in constitutive regulation of cellular events [32]. The receptor EP4 signals through increased cAMP [21] and is a systemic vasodepressor [33].

EP4 directs platelet inhibition at low PGE_2_ concentrations, but leads to EP3-mediated platelet aggregation at high concentration of PGE_2_[34]. It also has roles in ductus arteriosus closure and inflammation associated bone resorption [35]. The human EP4 cDNA encodes a 488-amino-acid polypeptide with a predicted molecular mass of 53 kDa [21]. EP4 may be pharmacologically distinguished from the EP1 and EP3 by their insensitivity to sulprostone and from EP2 by its insensitivity to butaprost [26,36].

DP: PGD_2_ is the major COX product released by mast cells during allergic responses. It has been associated with the development of pulmonary inflammatory diseases such as asthma [37]. PGD_2_ also plays a role in regulating sleep-wake cycles [38] and pain perception [39]. Two receptors for PGD_2_, DP1 [40] and DP2 [41], have been cloned from human cells. DP1 is coupled positively to adenylyl cyclase though Gs [14] and this results in strong inhibitory effects on platelet aggregation, bronchodilation and vasodilation in humans [42,43]. DP2 (also known as CRTH2) is preferentially expressed in T-helper type 2 cells, eosinophils, and basophils. Activation of DP_2_ leads to mobilization of intracellular Ca^2+^. DP2 also directs PGD_2_-induced chemotaxis and migration of TH2 cells [44]. Both DP1 and DP2 integrate coordinately the effects of PGD_2_ on eosinophils, modulating chemokinesis, degranulation, and apoptosis [45].

FP: Prostaglandin F_2α_ (PGF_2α_) is synthesized from PGH_2_ via a PGF synthase or from PGE_2_ via a 9-keto reductase. PGF_2α_ is the trigger that initiates luteolysis or regression of the corpus luteum in the absence of pregnancy [46]. It is also a contractor of smooth muscle across a variety of tissues [47]. PGF_2α_ induces cardiac myocyte hypertrophy and induction of myofibrillar gene [48]. Thus far, one membrane receptor for PGF_2α_ (FP) in human cells has been cloned [49]. Stimulation of FP activates Rho kinase, leading to the formation of actin stress fibers, phosphorylation of p125 focal adhesion kinase, and cell rounding [50]. FP is highly expressed in skin, where it may play an important role in carcinogenesis [51]. FP also appears to have an important role in the eye, where it increases uveoscleral outflow and reduces ocular pressure [52].

IP: Prostacyclin (PGI_2_) was first discovered in 1976 as an unstable eicosanoid in blood vessels [53]. It is very labile and undergoes spontaneous transformation to 6-keto-PGF_1α_ within minutes *in vivo*[54]. Prostacyclin is a potent endogenous anticoagulator for platelets and a strong vasodilator [53]. It is the most abundant product of arachidonic acid in vascular tissues [55]. Like many other lipid mediators of the eicosanoid family derived from arachidonic acid, PGI_2_ is produced by the COX system. PGI_2_ synthase (PGIS) converts PGH_2_ to PGI_2_[56]. On-site production of PGI_2_ is executed by either COX-1 or COX-2 coupled to PGIS [57]. However, PGI_2_ is a primary product of COX-2 in certain systems [58-60]. Prostacyclin signaling pathway involves both cell surface and nuclear receptors [61]. However, the classical signaling pathway of PGI_2_ is to use a G protein coupled receptor termed IP [62]. IP mRNA is abundantly expressed in kidney, liver, heart and lung [63]. Activation of IP by PGI_2_ leads to an increase in cAMP and activates protein kinase A cascade, or calcium mobilization via phospholipase C activation [64].

TP: Thrombosane A_2_ (TxA_2_) is produced from PGH_2_ by TX synthase. It is a modulator of platelet shape change and aggregation. TxA_2_ is a potent vasoconstrictor, mitogen and platelet activator [65-67]. TxA_2_ may also mediate cellular hypertrophy [68]. The human TxA_2_ receptor (TP) was the first eicosanoid receptor cloned in 1991 [69]. Two alternatively spliced variants of human TP have been described [70]. These variants differ in the C-terminal tail of the receptor distal to Arg-328. The original placenta-derived clone of 343 amino acid residues receptor has been designated as TPα, and a 407 residue splice variant cloned from endothelium is designated as TPβ. TP mRNAs are expressed widely in the lung, liver, kidney, heart, uterus, and vascular cells with TPα as the dominant isoform [71]. Both TPα and TPβ couple via G_q_, G_11_, and G_12/13_ to activate PLC-dependent inositol phosphate generation and elevate intracellular calcium [63]. Activation of TP by TxA_2_, or by more stable synthetic agonists, evokes the activation of phospholipase C and a subsequent rise in the intracellular calcium ion concentration, leading to vasoconstriction and platelet aggregation [72,73]. It is interesting to note that TPα and TPβ appear to dimerize, and their coexpression augments iPF2α-III (an isoprostane) signaling when compared to either receptor alone [74].

## Cyclooxygenases and cancer

Speculation of COX enzymes’ involvement in carcinogenesis and progression existed since 1976. That year, a small study showed that the osteolytic activities of 8 out of 9 osteolytically active tumors excised from patients with breast cancer were significantly inhibited by aspirin, a nonselective COX inhibitor [75]. Since then, aberrant over-expression of COX has been consistently associated with malignant transformation of healthy tissue, proliferation and increased invasiveness of malignant tissue, and unfavorable clinical outcomes. Up-regulations of COX enzymes have been reported in many human cancers and accumulation of PGE_2_, PGF_2α_ (a PGE_2_ derivative), and PGI_2_ are features of many epithelial cancers. COX-1 is up-regulated in cervical, ovarian and gallbladder cancers [1]. COX-2, which is normally undetectable in healthy tissue, is markedly over-expressed in colorectal, lung [76], prostate [77], cervical [78], ovarian [79], breast, gastric, pancreatic [80] and certain head and neck squamous cell [81] cancers. In the following paragraphs, we will examine the involvements of COXs in tumor initiation and progression in more depth.

## Cyclooxygenase involvement in tumor initiation

Studies in the laboratory are establishing COX enzymes’ roles in cancer initiation. Aberrant COX expression alone is not known to initiate tumors, but significant up-regulation of these enzymes is frequently associated with premalignant alterations in epithelial tissues. In addition to causing chronic inflammation, immunological studies have shown that COX products, especially Prostaglandin E_2_, interfere with the antitumor activities of the immune system. PGE_2_ inhibits T lymphocytes from producing antitumor T_H_1 cytokines and inhibit the antitumor functions of natural killer cells and macrophages [1]. Also, in tissues with low cytochrome P450 expressions, COX enzymes oxidize a significant amount of xenobiotics into mutagens and generate mutagens as by-products during prostaglandin synthesis [1]. Those endogenously produced mutagens can cause DNA damages as tumor initiators.

*In vivo* experiments have shown that APC^Min^ mice lacking COX-2 develop 80% fewer intestinal neoplasias than mice that express wild type COX-2 [82], and these COX-2 deficient mice also develop 75% fewer papillomas than wild-type mice in a multistep tumor initiation/promotion model [83]. Other studies have confirmed this correlation between COX-2 over-expression and premalignant and malignant lesions in epithelial tissues. Both pharmacological inhibition of COX-2 using celecoxib, a selective COX-2 inhibitor, and genetic knockout of COX-2 protected mice from UV-induced nonmelanoma skin cancers [84]. Mice that over-express COX-2 in basal epidermal cells, through keratin 5 promoters, are significantly more susceptible to genotoxic carcinogens than wild-type mice, and develop epidermal hyperplasia and dysplasia after single epicutaneous applications of 0.5 μM DMBA in 0.1 ml acetone [85].

Although there is strong evidence suggesting COX-2’s involvement in tumor initiation, COX-1’s functional role in carcinogenesis remains unclear – these same mice models have produced inconsistent data. While COX-1 deficient APC^Min^ mice also presented with an 80% lower incidence of intestinal neoplasia [82], COX-1 deficiency offered no protection against skin cancers in the UV-induced carcinoma model [84]. The studies suggest a potential role for aberrant expression and activities of COX-2, but less likely COX-1, in initiating tumor formation.

## Cyclooxygenase involvement in tumor growth and progression

Further studies have identified the COX enzymes as important components for tumor growth, survival and progression. Clinical surveys have found an average of a 3.3 fold difference between the expression of COX-2 enzymes in malignant prostate cancer tissues and inflammatory benign prostate hyperplasia tissues [77]. The same study also found marked increases of Bcl-2, an apoptosis suppressor, and vascular endothelial growth factor (VEGF), an angiogenesis modulator linked to accelerated tumor progression and increased invasiveness, in malignant prostate tissues [77]. *In vitro* studies have confirmed the correlation between COX-2 and many of these observations. Treatment with PGE_2_ receptor (EP) antagonists and siRNA silencing of COX-2 in Hela cervical cancer cell coincides with sharp decreases in VEGF-C protein and mRNA expression respectively [86]. Stromal fibroblasts isolated from COX-2 deficient, but not COX-1 deficient, mice also produces far less (>90%) VEGF than fibroblasts from wild-type mice [87]. Therefore, COX-2 appears to not only mediate tumor growth, but also it can elicit other changes from host tissues, such as neovascularization, to support tumor growth and progression.

Animal model experiments have echoed these findings. Although not statistically significant when compared to control groups, 25 mg/kg of body weight dose of celecoxib administered daily via gavage feeding tubes inhibited the growth of subcutaneously implanted SKOV-3 human ovarian carcinoma cells by 15% over 28 days in nude mice. Co-administration of celecoxib and 3 mg/kg SC-560, a selective COX-1 inhibitor, inhibited tumor growth by a statistically significant 36% over the same period of time [88]. Immunohistochemistry revealed significantly lower KI-67 expression in the tumors excised from all three (SC-560, celecoxib, and SC-560/celecoxib) treatment groups than the tumors from the control group [88]. TUNEL assays detected significant increases in fragmented DNA in the SC-560 or celecoxib treated groups, and a 167% increase, over the control group, in the SC-570/celecoxib group [88].

Another study confirmed that pharmacological inhibition of COX-2, but not COX-1, dramatically reduces cellular proliferation *in vitro*. Treatment of BxPC-3 cells, a COX-2 positive pancreatic adenocarcinoma cell line, with 25 μM or 50 μM NS-398, a selective COX-2 inhibitor, inhibited cell growth by 30% and 50% respectively [89]. Further, this study established a link between COX-2 expression and angiogenesis, the formation of new blood vessels that is required for tumor growth beyond 2-3 mm [1]. Pretreatment of BxPC-3 cells with 50 μM NS-398 inhibited BxPC-3 induced Bovine Aortic Endothelial Cell migration, a crucial step of the angiogenic process, by 68%; while treatment with 5 μM PGE_2_ after 50 μM NS-398 partially restored BAEC migration. Further, endothelial cells seeded onto Matrigels and co-cultured with BxPC-3 cells differentiated and developed into tube like structures *in vitro*, a phenomenon that is not observed in control groups lacking cancer cells or experimental groups that are continuously exposed to 50 μM NS-398. Mouse Matrigel plug assay, an *in vivo* angiogenesis model, further confirmed the angiogenesis inducing potential of COX-2 products. Mice injected with culture medium derived from BxPC-3 cells presented a 10 fold increase, over the control group, in neovascularization; versus only a 2.9 fold increase in mice injected with culture medium derived from AsPC-1, a COX-2 negative cell line. Furthermore, pretreatment of BxPC-3 cells with 50 μM NS-398 completely abrogated the derived culture medium’s ability to stimulate neovascularization in these mice [89].

There are numerous other studies in support of the anti-cancer activities of COX-2 inhibitors, most notably celecoxib. However, it should be noted that not all anti-cancer activities of celecoxib or other NSAIDs can be contributed to inhibition of COX activities and reduction of prostanoid biosynthesis. In fact, for example, the induction of apoptosis of celecoxib has been found not associated with inhibition of COX-2 [90]. As a small molecule, celecoxib can have off target activities, as evidenced by the observations that the acute cytotoxicity of celecoxib can be separated from its inhibition of COX-2. In addition to celecoxib, other NSAIDs can exert anti-proliferative or cytotoxic effects through various COX-independent mechanisms [91]. The studies raised interesting questions regarding various off target effects of NSAIDs including COX-2 selective inhibitors.

## Cyclooxygenase involvement in tumor invasion and metastasis

Given the involvement of COX-2 in cancer development and progression, clinical surveys have, not surprisingly, found strong positive correlations between high COX-2 expression and unfavorable clinical outcomes, especially metastasis [79]. The risk of death for patients with COX-2 positive ovarian cancer is 2.8 times that of the patients with COX-2 negative ovarian cancer – COX-2 expression, in this case, is a more predictive prognosis indicator than FIGO stage or histological grade [79].

COX-2 has been shown to promote invasive phenotypes through increased expression of matrix metalloproteinases (MMP) 1 and 2 to break down the extracellular matrix and decrease cell-cell adhesion, in human colon cancer cells and the hyaluronate receptor CD44, glycoprotein receptors involved in cell adhesion and migration, in colon [80] and non small-cell lung [1] cancers. Other studies have also confirmed that there is significantly higher COX-2 and VEGF-C expressions in lymph node metastasis (LNM) positive than in LNM negative cervical and lung cancers specimens [86]. Experiments have shown that *in vitro* siRNA knock-down of VEGF-C in A548 lung cancer cells partially restored epithelial phenotype, significantly decreased specific mesenchymal markers, and drastically decreased the side population of cells expressing cancer stem cell markers [92], which are speculated to contribute to the invasiveness and chemo- and radio-resistance in tumors [93]. Pharmacological inhibition of COX enzymes by NSAIDs has been shown to lower serum VEGF-C levels in patients. Celecoxib, when orally administered, lowers VEGF serum levels by as much as 15%-25% [94]. Orally administered 50 mg/kg rofecoxib, another selective COX-2 inhibitor, predictably decreased the incidence of liver metastasis in BALB/c mice carrying implanted MC-26 murine colon cancer cells in their splenic subcapsule. These treated mice also had a far lower mortality rate than the control group, 10% versus 90% after 30 days [95]. The studies generally support the rationale of using COX-2 inhibitor to reduce tumor invasion and metastasis. However, due to the adverse effects of long term use of COX-2 inhibitors, it is still unclear whether there is a therapeutic window of using COX-2 inhibitors in reducing tumor invasion and metastasis in a safe and effective way.

Among the downstream of COX, PGE synthase is extensively studied for its potential role in tumor progression and already subjected to a number of reviews [96-100]. Evidence accumulates suggesting another downstream enzyme, thromboxane (TX) synthase in tumor progression. TX synthase converts PGG_2_ to TXA_2_. Astrocytoma cells selected for migratory ability revealed that enhanced motility is coincided with up-regulated expression of a TX synthase homolog [80]. Increased expression of TX synthase was found in prostate cancer specimens with advanced stage and perineural invasion [101]. Further *in vitro* experiments have confirmed that up-regulation of TX synthase increases the migratory ability of DU-145 cells, a moderately invasive prostate cancer cell line, and SQ-29548, a potent and selective TXA2 receptor antagonist, interferes with the migratory ability of these cells [101]. NS-398 inhibited TXA_2_ synthesis *in vitro* by roughly 80%, and combination treatment with piroxicam, a selective COX-1 inhibitor, and NS-398 reduced TXA_2_ synthesis by 95% [101]. Therefore, in cancer cells, both COX isoforms can contribute PGH_2_ to form TXA_2_. Further studies of enzymes downstream of COX may provide target of intervention to reduce tumor invasion and metastasis as results of aberrant COX-2 or COX-1 expression and activities.

## Prostanoid receptors and cancer

There is even evidence of COX product dependence in malignant tissues that do not exhibit aberrant endogenous COX expressions. One study showed that COX-deficient pancreatic cancer cell lines AsPC1 and MiaPaCa2 undergo significant increases in cell proliferation after treatment with concentrations of PGE_2_ as low as 1 μM; and these cells showed markedly slowed growth after siRNA knockdown of MRP4, the main prostaglandin transporter [102]. This suggests that prostanoids are essential to cancer progression, and abnormally high concentrations of prostanoids, such as PGE_2_, in the cellular microenvironment, produced by non- or premalignant tissues that over-express COX enzymes, may affect cancer progression and clinical outcomes just as much as endogenous COX over-expression.

The receptors for prostanoids are involved in many pathophysiological processes, and they have been often linked to various diseases such as inflammation, atherosclerosis and cancer. For instance, it has been shown that prostaglandin E_2_ induced activation of EP4 mediates RCC7 cell invasion [103]. Specifically, EP4 that is expressed in renal cancer cells initiates a variety of signaling cascades that are transduced by activated Gα_s_. This signal transduction pathway further encompasses production of the second messenger cAMP by adenylyl cyclase and subsequent activation of Rap signaling that promotes cell migration [103].

Another study examined the role of EP receptors in invasiveness of breast cancer cells of murine (C3L5) and human (MDA-MB-231 and MCF-7) origin [104]. The major finding of this study was that breast cancer cells with high metastatic potential (C3L5 and MDA-MB-231) produced higher amounts of prostaglandin E_2_ than the noninvasive MCF7 cell line [104]. Moreover, the mRNA profiles for EP receptors were evaluated. It was observed that human cell lines expressed all four receptors but the murine cell line lacked expression of EP2 receptor. EP4 induced activation of the effector molecule PKA was implicated in an autocrine PGE_2_ migratory activity. Interestingly, the basal migration index of MCF7 (1.3+/−0.4%) cells was lower than those of MDA-MB-231 (28.1+/−6.4%), proving dissimilar migratory abilities [104].

These results are in close agreement with the implication of the EP1 signaling pathway in the local invasiveness and metastasis of oral squamous cell carcinoma [105]. This study provided evidence that PGE_2_ increases cell motility by activation of EP1, PKCδ, c-Src, c-Jun, and AP-1 signal cascade [105]. The transcriptional activity of c-Jun leads to expression of ICAM-1 that mediates adhesion-dependent cell-to-cell interactions and is known to facilitate movement [105].

Interestingly, another study demonstrated that decreased expression of EP1 at RNA level correlated with a poor prognosis in breast cancer patients [106]. Microarray analysis data suggest that the majority of tumors from European American women (72%) that showed significant levels of EP1 mRNA were correlated with a higher survival rate than African American women who expressed very low levels of EP1 mRNA [106]. Nuclear localization of EP1 had a beneficial impact on survival [106].

Evidence suggests that EP2 mediated signaling plays a pivotal role in the proliferation and apoptosis of human hepatoma cell lines (HepG2 and SMMC-7721) [107]. Results indicated that expression of EP2 directly correlated with elevated expression of the anti-apoptotic protein Bcl-2 [107]. In contrast, paeoniflorin induced apoptosis in liver cancer cells by suppressing expression of EP2 and augmenting Bax and cleaved caspase-3 levels [107].

Furthermore, the biological capability of prostaglandin E_2_ to induce EP4 activation and metastasis of breast cancer cells involves a mechanism that requires natural killer cells [108]. NK cells have been demonstrated to express all four EP receptors, but preferential activation of EP4 by PGE_2_ is responsible for inhibition of NK cell migration and cytokine secretion [108]. The compromised function of NK cells contributes to suppression of critical NK cell functions involved in control of metastasis [108].

Recent studies have demonstrated that PGD_2_ coupling to DP receptor promotes inhibitory effects on NK cell functions [109]. The considerable clinical implication in cancer derives from the fact that DP is expressed on human NK cells and PGD_2_ mediated activation of this receptor leads to inhibition of the cytotoxic and chemotactic effects as well as decreased accumulation of type 1 cytokine. Intriguingly, this inhibition prevents NK cell migration toward the inflammatory site [109]. A low dose (10nM) of PGD_2_ is sufficient to inhibit NK functions. This anti-inflammatory response may prove to be beneficial in cancer treatment [109]. The therapeutic potential of DP agonists has also been demonstrated by the fact that DP deficiency enhances tumor progression and angiogenesis [110]. Specifically, DP expression in endothelial cells decelerates vascular leakage which results in decreased tumor angiogenesis and tumor growth [110]. Further, it has been speculated that both aldo-keto reductase AKR1C2 and AKR1C3 mediated PGD_2_ catabolism enhanced prostate cancer cell proliferation via FP and PI3K/Akt signaling pathways [111].

Studies have highlighted the mechanism of FP receptor signaling in alteration of epithelial cell invasion and endothelial cell function in endometrial cancer [112]. Ligand (PGF_2a_) induced activation of FP in the epithelial cells of endometrial adenocarcinoma, results in stimulation of the calmodulin-NFAT signaling pathway [112]. This signaling cascade leads to elevated ADAMTS1 which functions in an autocrine/paracrine manner to promote epithelial cell invasion via ECM and a paracrine manner to inhibit endothelial cell proliferation [112]. The same group also suggested the presence of a positive feedback loop that regulates neoplastic epithelial cell function in endometrial adenocarcinoma [88]. Specifically, COX-2 induced production of PGF_2a_ stimulates its binding to the FP receptor and subsequent G_q_ and 1,4,5-triphosphate activation [88]. The later results in stimulation of ERK signaling and subsequent enhanced production of fibroblast growth factor 2 (FGF2) and expression of additional COX2. FGF2 association with FGFR1 promotes phosphorylation of ERK and enhanced cellular proliferation [88].

Further, it has been proposed that PGF_2a_ – FP association potentiates angiogenesis in endometrial adenocarcinoma through activation of EGFR and subsequent ERK1/2 signaling. This resulted in enhanced signaling and transcription of the vascular endothelial growth factor (VEGF) [113].

Expression of the prostacyclin receptor IP is indicative of the angiogenic phenotype of tumor endothelial cells according to findings that suggested that migration and tube formation of TEC were inhibited by the IP receptor antagonist RO1138452 [102]. According to the same study, IP receptor is required for induction of angiogenesis. It has been speculated that stable prostacyclin analogues reduce lung and lymph node metastasis in mammary carcinoma models [114].

It has been reported that thromboxane A_2_ receptors (TXA_2_R, or TP) play a pivotal role in cell transformation and proliferation of neoplastic human lung cells through expression of Nurr1 [115]. Nurr1 an orphan nuclear receptor has been identified as the downstream effector of TP signaling. Agonist (I-BOP) induced activation of TP and stimulation of Nurr1 expression was mediated via the PKA/CREB, PKC and ERK signaling pathways in H157 cells [115]. Nurr1 is believed to mediate cyclin D1 expression and cell proliferation. Moreover, constitutive interaction between TPα/TPβ and PRK1 leads to phosphorylation of histone H3 at Thr [11] and associated cell migration and proliferation in prostate carcinoma cell lines [116]. It has been noted that TPβ could be used as a predictor of prognosis for bladder cancer, since bladder cancer cell lines express this isoform unlike SV-HUC that exclusively express TPα [117]. TPβ expression positively correlates with cell proliferation, migration and invasion in bladder cancer [117]. In prostate cancer, activation of TP could lead to cytoskeletal reorganization and rapid cell contraction through activation of small GTPase RhoA [118]. Blockade of TP activation compromised tumor cell motility [118].

Despite the above described studies indicating the involvement of prostanoid receptors in cancer (Figure 1), it still remains to be defined when and how prostanoid receptors are enlisted during tumor formation and progression, and whether prostanoid receptors can be a target of intervention for cancer prevention and treatment.

## Targeting cyclooxygenases for cancer prevention and treatment using NSAIDs

Experiments in the laboratory have indicated that COX inhibitors are promising candidates that could be used to treat cancer. So far, COX inhibitors have been shown not only to inhibit tumor initiation and accelerated progression, but also to preferentially affect cancer cells. At least one study has reported that *in vitro* 100 μM NS-398 treatments markedly reduced the viability of LNCaP, an androgen-sensitive human prostate adenocarcinoma cell line, but not the viability of human fetal prostate fibroblasts. Analysis of the genomic DNA extracted from these cells also showed that only malignant cells experienced increases in DNA fragmentation, 11 folds to be precise, after three days treatment [119].

These findings, however, have not been translated successfully into the clinics. Initial findings show that COX inhibitors are ineffective at preventing polyp growth in patients with familial adenomatous polyposis coli (FAP) at the anti-inflammatory doses, but long term use of these drugs at higher doses are associated with serious side effects. Nonselective COX inhibitors are often associated with gastrointestinal problems, including serious, albeit rare, gastric and duodenal perforations, and selective COX-2 inhibitors are associated with significantly increased risk of acute myocardial infarctions in already at-risk patients [1], although more recent studies show that celecoxib can be safely administered at 800 mg/day in combination with other treatments [120].

So far, clinical trials involving treatment regiments that target the COX pathway have produced mixed results. While, low dose celecoxib used in combination with temozolomide, a DNA methylation/alkylation agent often used to treat glioblastoma multiforme and melanomas after gross surgical resection and radiotherapy, does prolong progression free survival for patients with glioblastoma multiforme, it also, rather counter-intuitively, increases the likelihood of distant metastases by 3 to 12 folds [121]. Orally administered 400 mg celecoxib twice a day decreased the KI-67 index in bronchi tissues extracted from former smokers by an average of 34% over six month, compared to only 3.8% in the placebo group [122], while the same dose taken in combination with palliative chemotherapy for patients with non-small cell lung cancer (NSCLC) provided no survival benefit over the placebo [123]. Patients who take selective COX-2 inhibitors for one year or more are at a lower risk for colorectal cancers, but at a significantly higher risk for breast and hematological cancers [124]. These mixed results make it necessary to further elucidate the mechanism of actions of NSAIDs in reducing cancer incidence and/or mortality so that a subset of patients can be identified for targeted use of NSAIDs for cancer prevention or treatment.

## Targeting the prostanoid signaling pathways for cancer treatment

Long-term administration of patients with COX-2 selective inhibitors may have consequences such as severe cardiovascular complications. Due to this major drawback of the current therapeutic strategies aiming at inhibition of cyclooxygenase activity, efforts are currently underway to discover administration of prostanoid signaling pathway antagonists.

Prostacyclin (PGI_2_) is the main product of arachidonate metabolism and has pleiotropic biological activities such as vasodilation. Its role as an endogenous inhibitor of platelet aggregation has been further investigated as a beneficial activity to reduce tumor metastatic process [125]. Specifically, a study indicated that lungs treated with PGI_2_ had 40–50 times fewer metastatic nodes when compared to the positive control [126]. The same study showed that treatment of mice with PGI_2_, resulted in a 10% decrease of metastatic cell adhesion to the endothelial tubules [126]. An alternative approach for the therapeutic targeting of prostanoid signaling, was the examination of the inhibitory effect of COX selective inhibitors on prostacyclin. A significant inhibition of prostacyclin synthase activity was observed in human endothelial cells, following treatment with rofecoxib [127]. Currently a plethora of prostacyclin receptor (IP receptor) agonists such as iloprost, cicaprost and carbacyclin have been reported. However, only a few highly selective IP receptor antagonists such as 2-[4-(1*H*-indol-4-yloxymethyl)-benzyloxycarbonylamino]-3-phenyl-propionic acid are known [128]. It still remains to be elucidated when and how PGI_2_ and its receptor can be exploited for cancer treatment.

Reports regarding the major COX-2 metabolite PGE_2_ and its association with the development of colorectal cancer and other malignancies through its cognate receptors have been extensively published. Experimental evidence supports that ONO-8711, an E-prostanoid receptor antagonist, inhibits PGE_2_ signaling without any interference on the PGF_2a_ production or PGIS expression [129]. The fact that it has no effect on prostacyclin production might render ONO-8711 as a safer chemopreventive agent regarding cardiovascular events [129]. Another study showed that indomethacin, a major NSAID, antagonizes human EP2 receptors. The unfavorable lack of efficacy and specificity of indomethacin led to the consideration of other therapeutic agents [130]. Epigallocatechin-3-gallate, a naturally occurring dietary compound extracted from green tea has been identified as a potent suppressor of cellular PGE_2_ biosynthesis [131]. The same substance has been shown to synergistically augment celecoxib-mediated suppression of PGE_2_ as well as decrease the amount of the COX-2 inhibitor (celecoxib) necessary for the production of the anti-tumoral effect [132].

Another eicosanod studied as a target for cancer prevention and treatment is the thromboxane A_2_ formed by the action of thromboxane A_2_ synthase (TXA_2_S). An approach that has been investigated is the inhibition of TXA_2_S, which involves selective suppression of thromboxane formation. Dazoxiben is an example of this class of drugs and prevents the conversion of PGH_2_ to TXA_2_. This drug shifts the direction of metabolism towards the production of PGI_2_ and PGD_2_[133]. Researchers tested the effects of administration of pirmagrel, a thromboxane synthetase inhibitor in 10 renal allograft recipients with cyclosporine nephrotoxicity and found that it effectively suppresses the production of TXA_2_ as indicated by the reduction in the levels of the inactive TXB_2_ (96% mean suppression) and other thromboxane derived metabolites [134]. These results are in close agreement with decreased levels of TXB_2_ in serum during UK-37 248 TXA_2_S inhibitor administration. In addition, the imidazole derivative UK 37 248 increased the production of PGE_2_ and PGF_2a_[135]. Another TXA_2_ synthase inhibitor OKY046 inhibits not only the production of TXA_2_ but also its release without increasing PGI_2_ synthesis [136]. Remarkably, E3040, a dual inhibitor of 5-lipoxygenase and thromboxane synthase at 30 and 100 mg/kg inhibited the synthesis of leukotriene B4 and thromboxane B2 but increased the production of PGE_2_[137].

Further strategies have focused upon the direct blockade of the TP receptor. SQ29548 is a standard TP antagonist that has contributed to the investigation of TP mediated processes. Several data have indicated that domitroban (S-1452/S-145) which is a TP antagonist, is more potent than OKY046 (TXA_2_S inhibitor) and is capable of hampering cytokine synthesis [138]. Ramatoroban (Bay U3405) is a potent long lasting inhibitor of U46619 (TXA_2_ agonist). Research has shown that it is effective after either intravenous or oral administration in male Dunkin Hartley guinea pigs with ID_50_ values of 600 ug/kg and 1.7 mg/kg respectively [139]. Extensive studies have shown that combined administration of aspirin with TP antagonist apigenin, essentially potentiates the inhibitory effect on the cyclooxygenase TXA_2_ pathway in the instance of aspirin failure to properly suppress the TXA_2_ pathway [140]. Other flavonoids such as genistein and luteolin have been shown to displace binding of radiolabeled SQ29548 by >50% to different cell types [141]. Several lines of evidence suggest that a superior method for the reduction of TXA_2_ synthesis is the development of drugs that have dual functions as TP antagonists and thromboxane synthase inhibitors. The category of dual acting drugs includes: picotamide (G137), samixogrel (DTTX30) and BM-531 [142].

Most inhibitors of prostanoid signaling are developed for cardiovascular indications or as anti-inflammatory agents. Clearly more studies are needed to define the involvements of prostanoids in tumor formation and progression and to evaluate the utility of these proostanoid inhibitors in cancer prevention and treatment.

## Conclusion and perspective

COX enzymes clearly become deregulated in cancers and all research indicate that these metabolic pathways are involved in carcinogenesis and tumor progression. Studies using animal models have demonstrated that these enzymes are promising targets for intervention; *in vivo* models consistently show that COX inhibitors inhibit tumor incidence, growth, and acquisition of invasive phenotypes. Unfortunately, these results have not yet been translated into the clinics successfully.

COX expression is indicative of poorer prognosis for cancer patients, but the biological effects of its metabolites are diverse and often in opposition. Most studies indicate that the ultimate fate and biological effect of PGH_2_ is largely tissue dependent, blind systemic inhibition of COX enzymes risks upsetting the delicate COX metabolite homeostasis, so it’s not surprising that clinical trials have produced largely inconsistent results. Groups, however, have independently confirmed that selective inhibition or deletion of specific PGE_2_, PGF_2α_, TXA_2_ receptors produces many of the same effects as direct COX inhibition [143-146]. Further research into these specific downstream pathways should be conducted, since more targeted treatments are more likely to yield desirable results.

There is no complete account of how COX and LOX enzymes become deregulated in cancer. Even though the demand for this answer might be considered unreasonable, especially since many other unknowns that are far more likely to yield immediate therapeutic options still exist. This question, however, is scientifically intriguing and an answer is necessary for a holistic understanding of cancer biology. Studies have already reported that co-administration of COX and LOX inhibitors produces a synergistic effect. More recently, studies have reported that NSAID induced apoptosis coincides with increased 15-LOX expression and activity [147,148] and that celecoxib treatment may affect LTB4 levels in lung, colon and prostate cancers [149,150] So far, evidence only hint that these pathways are connected or operate in concert. More research should be conducted to clarify this connection.

Finally, LOX isozymes oxygenate a diverse set of PUFAs, not just AA. Metabolites generated from other substrates often exert effects that oppose those of the AA metabolites. Studies have found correlations between dietary ω-6 fatty acids and cancer [151], and at least one study has reported that dietary ω-3 fatty acid supplements enhance anti-VEGF and COX inhibitor thearpies [152]. Research into this field can have profound impact on our understanding of nutrition and identify diets that supplement and enhance existing treatments.

## Competing interests

The authors do not have any competing interests to declare.

## Authors’ contributions

VA drafted the portion of prostanoid receptors and cancer, CG drafted the portion of cyclooxygenases and cancer, DN revised the whole manuscript and approved the manuscript. All authors read and approved the final manuscript
